# Leading, Connecting and Coping: Nurses’ Responses to Sustaining Well‐Being During the COVID‐19 Crisis—A Qualitative Study

**DOI:** 10.1155/jonm/8830477

**Published:** 2026-08-29

**Authors:** Pauline Calleja, Jenny Kelly, Stephen Yu, Colleen Ryan, Matthew Hiskens, Burton Linda Roszak, Lynette Graham, Marree Porch, Caroline Laker, Pamela Knight-Davidson, Michelle Cleary

**Affiliations:** ^1^ College of Healthcare Sciences, James Cook University, Townsville, Queensland, Australia, health.qld.gov.au; ^2^ School of Nursing, Midwifery & Social Sciences, CQUniversity, Rockhampton, Australia, cqu.edu.au; ^3^ School of Nursing & Midwifery, University of Southern Queensland, Toowoomba, Australia, usq.edu.au; ^4^ Mackay Hospital and Health Service, Mackay, Queensland, Australia, health.qld.gov.au; ^5^ College of Medicine and Dentistry, James Cook University, Townsville, Queensland, Australia, health.qld.gov.au; ^6^ DRW Coaching, Baltimore, USA; ^7^ Cairns and Hinterland Hospital and Health Service, Cairns, Queensland, Australia, health.qld.gov.au; ^8^ School of Health & Psychological Sciences, University of London, London, UK, london.ac.uk; ^9^ Faculty of Health, Education, Medicine and Social Care, Anglia Ruskin University, Chelmsford, UK, anglia.ac.uk; ^10^ School of Nursing & Midwifery, University of Technology, Sydney, New South Wales, Australia, uts.edu.au

**Keywords:** mental health, nurses, organisational crisis, public health, support strategies, well-being

## Abstract

**Aim:**

To explore nurses’ perceptions of the strategies used to support their mental health and well‐being during a recent public health crisis.

**Design:**

A pragmatic, qualitative, exploratory design was employed.

**Methods:**

Semistructured interviews were conducted with a purposive sample of 20 nurses working in two large, regional public hospitals and health services in Queensland, Australia, between July and October 2023.

**Results:**

Three main themes were constructed from the data: (i) management and leadership approaches, (ii) connection to others and (iii) self‐care practices. Eight subthemes support the three themes.

**Conclusion:**

Although nurses and healthcare leaders could not control the broader impact of the crisis, the prolonged and evolving nature of the situation brought significant challenges to workforce management. Amidst the uncertainty, adaptability emerged as essential. The crisis also created space for innovations in flexibility and revealed the central role of peer support and teamwork in coping. Moreover, the importance of self‐care, particularly through maintaining boundaries between professional and personal life, was widely recognised as crucial to sustaining well‐being.

**Implications for Nursing Management:**

Nursing management has an important role in sustaining staff well‐being during prolonged crisis conditions. Given the centrality of peer support and teamwork as coping strategies, nursing managers should prioritise team cohesion through structured opportunities for connection and debriefing. Organisational approaches should also move beyond short‐term gestures and embed accessible, proactive well‐being supports that enable boundaries and recovery over time.

**Reporting Method:**

The study adheres to the COREQ reporting guidelines.

**Patient or Public Contribution:**

Participants in this study were registered nurses employed in two regional public hospitals in Queensland, Australia. Participants shared their experiences through in‐depth interviews. Each participant provided informed consent before taking part in the study.

## 1. Introduction

The unprecedented challenges of the recent COVID‐19 pandemic have alerted us to the critical importance of workplace well‐being, particularly for frontline clinical staff [1]. It has highlighted the focus on the challenges and stressors healthcare professionals often experience in their practice, such as burnout, compassion fatigue [2] or healthcare staff fatigue [3], and a lack of support for staff in their workplaces [4]. As a result of work pressures due to the public health crisis, nurses and other caregivers were predisposed to mental and emotional exhaustion [5], increased risk of burnout [6] and nonfatal suicidal behaviour [7]. At a time when staff recruitment and retention in the nursing workforce are vital [8, 9], there is a genuine concern about how best to support nurses and other healthcare professionals to cope with the potentially harmful effects associated with their roles in providing healthcare services [10].

Research on the prevalence of mental health problems since the emergence of the pandemic has lacked sensitivity across different contexts and has not necessarily included outcomes meaningful to the nursing workforce [11–13]. To effectively manage patient and workforce outcomes, healthcare organisations need to implement mental health and well‐being practice strategies that encourage self‐care and a supportive workplace [14]. Beyond Blue, a renowned mental health support organisation in Australia, recommends a four‐step process to develop workplace mental health and well‐being strategies [15], including (1) gaining leadership support, (2) analysing the situation, (3) establishing an action plan and (4) monitoring, reviewing and improving. An important influencing factor in these strategies is the organisational leadership. A recent review [16] underscored the critical role of strong leadership in fostering the mental health and well‐being of the nursing workforce through practical support strategies, especially during a public health crisis. It is, therefore, more important than ever that healthcare organisations ensure strategies are in place to improve the management of staff well‐being.

This study explored participants’ experiences and views of the strategies used to support their mental health and well‐being during the recent COVID‐19 pandemic. Lessons learned from this study may be used by health professionals and healthcare organisations to adequately prepare them for managing the mental health and well‐being of staff during future public health crises. Further research is needed to explore how organisations and health professionals work collaboratively to prepare staff to manage mental health and wellbeing during times of crisis.

## 2. Background

The critical role of frontline clinical staff, particularly nurses, in healthcare systems was unequivocally underscored during the COVID‐19 pandemic. It highlighted the significant workplace stressors that the healthcare professionals endured, with many reporting a lack of adequate support within their work environments [10, 12, 17], including neglect in the support for graduate nurses [18, 19]. These factors not only compromise individual well‐being but also have detrimental consequences for the nursing profession, manifesting as job dissatisfaction, increased intention to leave [9], diminished compassion [20] and compromised quality of patient care [21]. Consequently, addressing the mental health and well‐being of nurses has rightfully ascended to the forefront of the healthcare agenda [10].

While Australia experienced comparatively fewer actual COVID‐19 cases [22], the workload associated with preparedness and the reported stress levels among Australian nurses have been similar to those reported by their international counterparts managing higher caseloads [12]. Traditionally, approaches to managing stress and promoting emotional coping strategies have often focused on individual resilience‐building [2, 23] or group support forums [23]. However, while individual coping mechanisms can mediate the impact of workplace stressors, this approach has been criticised for placing the onus of psychological well‐being solely on the individual [23]. Furthermore, research suggests a complex relationship between mental health states and resilience levels [24]. This underscores the importance of acknowledging organisational responsibilities [25, 26] and the influence of the broader workplace ecosystem.

There is also evidence in the literature for employers to prioritise the mental health and well‐being of healthcare workers [27, 28]. An example of significant levels of stress, anxiety and depression is evidenced among critical care nurses [29], including the occurrence of medical errors [30]. These impacts are detrimental not only to individual nurses but also to the quality of care they provide [20, 21], as well as patient safety and workforce sustainability [14]. Consequently, there is a growing interest in the sustainable, system‐wide adoption and implementation of mental health support and stress reduction strategies. These approaches recognise the risks to mental health and well‐being as inherent occupational hazards that interact with personal and home life demands [13, 14]. Integral to this shift are ‘whole system’ and codesigned approaches that actively incorporate staff input regarding their local needs and contexts and engage management at all organisational levels in fostering staff well‐being [31–33].

This study addresses a recognised gap in the literature by exploring nurses’ experiences of the organisational and leadership strategies used to support mental health and wellbeing during the COVID‐19 pandemic. By moving beyond a focus on the prevalence of psychological distress, it provides practical insights into the workplace supports, leadership practices and organisational approaches that nurses perceived as valuable in real‐world clinical settings. The study was designed to create actionable evidence for nursing managers and healthcare organisations seeking to develop context‐specific, system‐level wellbeing strategies that strengthen workforce support, retention, resilience and preparedness for future health crises.

## 3. The Study

### 3.1. Study Aim

The aim of this study was to identify and explore nurses’ perceptions of the strategies used to support their mental health and well‐being during a recent public health pandemic.

## 4. Methods/Methodology

### 4.1. Study Design

A pragmatic, qualitative, exploratory design was used. This design is well documented to support the development of rigorous and contextually relevant interventions in complex settings [34] where descriptive or exploratory work must act as a prelude to intervention work [35]. The pragmatic approach encourages researchers to choose methodology/methods to best answer research questions rather than adhering to traditional approaches [36].

The reporting of this study adheres to the Consolidated Criteria for Reporting Qualitative Research (COREQ) [37].

### 4.2. Study Setting and Recruitment

Two publicly funded, regional Queensland hospital and health services were selected as study sites. Both sites, referred to as site A and site B, are located in outer regional areas according to the Australian Standard Geographic Classification, Accessibility/Remoteness Index of Australia Plus (ARIA+) [38].

Site A provides services to approximately 250,000 people. It is the only major referral hospital in the region, providing the most specialised health services. However, a few high‐level acute services are offered at two other major centres, located 347 and 1,700 km away. In addition to the major hospital, site A includes several smaller hospitals and Primary Health Care Centres, serving extremely remote communities in the outer western region.

Site B serves approximately 180,000 people across various regional, community and rural settings. The main hospital acts as the referral hospital for the region. Alongside the main hospital, site B comprises several smaller hospitals and community health facilities.

Purposive sampling was employed [39] to ensure the greater balance of nurses interviewed were direct care providers and that each site sampled at least three nurses in management roles. Specific criteria were used to select participants from those who expressed interest and included:

• New graduate nurses providing patient care (≤ 1 year of clinical experience)
• Registered and enrolled nurses with ≥ 1 year of clinical experience
• Nurse educators
• Nurse managers (NMs), including Nursing Directors and Executive Directors of Nursing and Midwifery
• Must be employed at least 0.6 full‐time equivalent (FTE) at the site

The final sample comprised 20 registered nurses (RNs): site A (*n* = 9) and site B (*n* = 11) (Table 1). This sample was within the planned sample of 18–24 nurses. Identifying upper and lower limits of a sample for qualitative interviews is supported in pragmatic qualitative research as opposed to data saturation as a concept to determine sample size [40]; however, many methodologists identify that this sample size does achieve saturation [41], and saturation did occur in this study within this sample size.

**TABLE 1:** Participant demographics.

Participant number and pseudonym	Gender	Role: registered nurse (RN)/nurse manager (NM)	Site A or B
NM 1 Stella	F	NM	A
NM 2 Crash	M	NM	A
NM 3 Bob	M	NM	A
RN 1 Claire	F	RN	A
RN 2 Holly	F	RN	A
RN 3 James	M	RN	A
RN 4 Kim	F	RN	A
RN 5 Lee	F	RN	A
RN 6 Natasha	F	RN	A
NM 1 Bethany	F	NM	B
NM 2 Ivy	F	NM	B
NM 3 Jane	F	NM	B
NM 4 Nicole	F	NM	B
NM 5 Terri	F	NM	B
NM 6 Yvonne	F	NM	B
RN 1 Amanda	F	RN	B
RN 2 Ashley	F	RN	B
RN 3 Emily	F	RN	B
RN 4 Kath	F	RN	B
RN 5 Susie	F	RN	B

### 4.3. Data Collection

At each participating site (site A and site B), the principal investigator and a designated coinvestigator served as site leads, overseeing research coordination. Two research assistants (one per site), who were not part of the recruitment team, conducted the semistructured, in‐depth individual interviews, ensuring participants were interviewed by an independent third party with no ties to the management of the participating health services or the original research team. Interviews were conducted either face‐to‐face or via Zoom/Teams.

To ensure consistency while allowing flexibility, a semistructured interview guide (Appendix A) was developed for in‐depth exploration of key issues. Members of the wider research team collaboratively designed and finalised the guide, ensuring alignment across interviews conducted by different interviewers.

Interviews were recorded with participant consent, and all data were deidentified. To enhance accuracy, research assistants validated key information in real time by summarising main points during the interview.

### 4.4. Data Analysis

Inductive thematic analysis [42] was used to code and analyse the data. This process was led by the principal investigator. Due to ethical restrictions, only the two site leads had access to identifiable data. However, once interviews were recorded and transcribed, they were no longer identifiable.

Guided by the principal investigator, research assistants coded the data using an established framework for reflexive thematic analysis. In line with this approach, the Australian researchers on the team employed inductive methods for coding and theming [40, 43]. This included the steps of reading and re‐reading the data (data familiarisation), assigning codes to data using participant terminology where possible (generating initial codes) and gaining consensus between researchers on code groupings (constructing initial themes). Themes were collated by the principal investigator and organised in an Excel spreadsheet, supported by participant quotes; definitions and naming themes were reached through group consensus. The international researchers, along with the Australian researchers, reviewed the coded data and contributed to interpreting and discussing the results. These last steps concluded the process of reviewing and reining themes, dinging and naming themes and writing up themes into a coherent narrative [40, 43].

Consensus was reached through a reflexive and iterative process, where team members independently reviewed coded data, compared interpretations and engaged in structured discussions. Disagreements were resolved through negotiated agreement, ensuring coherence and validity in theme identification.

### 4.5. Ethical Considerations

Ethical approval for the study was granted by the state government funded health service Human Research Ethics Committee (87982) and the University Human Research Ethics Committee (24078). All governance procedures for both sites were followed. Participants provided written or verbal informed consent prior to participation in the study. Participants were asked to choose a pseudonym and were informed they could terminate the interview at any time without any repercussions from their employer.

### 4.6. Rigour and Reflexivity

The study employed a pragmatic, qualitative, exploratory design with semistructured interviews conducted across two regional public hospitals. To ensure credibility, the researchers used systematic thematic analysis and maintained an audit trail of coding decisions. Dependability was supported by consistent data collection procedures and documentation of contextual factors.

The research team was made up of mostly nurses, two who are clinicians, and two team members working in health adjacent roles as health researchers. Two team members counted mental health as their nursing specialty area, and five have worked extensively in workforce wellbeing. The lead researcher had research and clinical experience in workforce wellbeing, regional nursing practice and nursing management experience. Three team members were employed by the health partner research sites, and the lead for each site was academic team members, not employed by the health service who lived locally in the health service catchment area.

Potential biases included local context biases for those researchers employed by the health service, researchers who had clinical or management experience potentially pre‐empting what the challenges would be with well‐being post‐COVID‐19 due to their own lived experience, and then the usual biases present related to any research design (e.g., selection/participant bias, analysis bias etc.) [44]. To manage these biases, the research team engaged in ongoing reflexive practice throughout the study. This included maintaining reflective field notes to examine their own assumptions, positionality and potential influence on data interpretation. Team discussions were held regularly to challenge interpretations and ensure that findings remained grounded in participants’ voices rather than researcher bias. Design and implementation of this study was adjusted specifically to ensure minimisation of bias where possible by

• employing data collectors who were not on the research team or employed by the health service,
• selecting participants based on pre‐exiting criteria,
• multiple data coders (three) who were not employed by the health service,
• multiple data points to validate and triangulate theme creation,
• understanding of meaning was built into the structured discussion guides by training the data collectors to summarise and paraphrase main points made by participants at the end of the interview,
• during the data analysis process looking for disconfirming evidence and contradictions.

These strategies helped to unite a diverse and geographically dispersed research team to manage potential biases in this study.

## 5. Findings

Twenty RNs employed in two, large, regional public hospital and health services in Queensland, Australia, participated in a semistructured interview between July and October 2023. Interviews average length was 30.01 min. Demographic data are presented in Table 1. Three main themes were constructed from the data: (i) management and leadership approaches, (ii) connection to others and (iii) self‐care practices. Each main theme consisted of a number of subthemes (Table 2).

**TABLE 2:** Themes and frequency contribution.

Themes	Sub‐themes	Participant contribution
Management and leadership approaches	• Extended crisis mode	Site A: NM‐1, 2, 3. RN‐1, 3, 4, 5, 6. Site B: NM‐1, 2, 3, 4, 6. RN‐1, 2, 3, 5.
	• Forced to be flexible	Site A: NM‐1, 2, 3. RN‐1, 2, 3, 5, 6. Site B: NM‐1, 2, 3, 4, 5. RN‐1, 2, 3.
	• Perceived divide: us and them	Site A: NM‐1, 2. RN‐2, 5, 6. Site B: NM‐6. RN‐1, 3.
	• Consultative, collaborative style	Site A: NM‐2. RN‐1, 2, 3, 5, 6. Site B: NM‐1, 2, 4, 5, 6. RN‐1, 2, 5.

Connection to others self‐care practices	• Support from peers	Site A: NM‐1, 2, 3. RN‐2, 6. Site B: NM‐1, 2, 3, 4, 5, 6. RN‐1, 2, 4, 5.
	• Teamwork	Site A: NM‐1, 3. RN‐1, 2, 5, 6 Site B: NM‐1, 2, 3, 4, 6. RN‐1, 2, 3, 5.
	• Self‐enriching activities	Site A: NM‐1, 2, 3, 5. RN‐ 1, 2, 3, 4, 5, 6. Site B: NM‐1, 2, 3, 4, 5, 6. RN‐ 1, 2, 3, 4, 5
	• Boundary setting	Site A: NM‐1, 2, 3. RN‐1, 2, 3, 4, 5 Site B: NM‐1, 4, 6. RN‐1, 3, 4, 5

### 5.1. Management and Leadership Approaches

#### 5.1.1. Extended Crisis Mode

Participants described varying approaches mediated by leadership style and proximity to the workforce. While some were highly critical of managements’ crisis‐driven and reactive focus, others acknowledged the significant demands they faced. During the crisis, the focus was heavily on service delivery rather than the well‐being of the teams. Some participants described that line managers went into self‐protective mode, leaving staff feeling unsupported and responsible for taking action to support their colleagues. Despite various strategies being implemented, there was a sense of uncertainty and a reactive approach to managing the extended crisis.

We were very much in a crisis mode, and I believe that we focused a lot more on service delivery than we did on the well‐being about teams [Crash, A‐NM2].

The staff were committed to ensuring the facility remained operational and open, but many found that support was lacking. Staff members had multiple additional responsibilities, which left them with little time to consider the well‐being of their colleagues.

They [management] also wear multiple hats … they have their fingers in a lot of pies and that means that their job overall, they don′t really have time to slow down and sort of take into consideration the well‐being and stuff of the staff [Natasha, A‐RN6].

#### 5.1.2. Forced to Be Flexible

Participants highlighted various workplace changes introduced in response to the ever‐evolving disaster situation. Flexible strategies regarding workforce management and clinical care were implemented at both sites and were generally well received by nursing staff. However, these strategies led to a mix of positive and negative outcomes.

We were given a bit of rope to do what we could do within our space as long as we weren’t spending any more money or requesting any extra resources it really didn’t matter. It helped a little [Bob, A‐NM3].

… we became a little bit more rigid in that we had clinic days, we had a home visiting days, whereas before it was all a bit wishy washy, you could do what you wanted [Claire, A‐RN1].

#### 5.1.3. Perceived Divide: Us and Them

Several participants noted a perceived division between frontline clinicians who worked closely with patients and managers who seemed distant and detached from the rapidly evolving clinical environment. This most often was referred to as executive management rather than immediate clinical line managers. While some nurses expressed feelings of being dismissed and let down and therefore not supported by management, others acknowledged that managers were also navigating the challenges of an uncertain and swiftly changing situation.

The entire time we were just told by the managers when we kept asking for support … ‘just get on with it’. You guys know what you’re doing and you′ve got lots of experience… So that was pretty disappointing [Stella, A‐NM1].

… those emails every other day from the Director General and from our ED [Executive Director] head Dr or like, this is what we′re doing now … And from now on, everyone must do this. Which I don′t think you can really help, but it gets a bit like you know, you′re a kid, being told what to do by your parents every day and they kind of had to, [be] ‘cause it was always evolving day by day’ [Holly, A‐RN2].

#### 5.1.4. Consultative, Collaborative Style

Managers who were supportive, involved staff in discussions and decision‐making and were highly regarded and appreciated by clinicians. This inclusive approach empowered and supported staff, fostering a positive and collaborative work environment. This was most often from immediate line managers rather than executive or higher level management.

It was an empowering approach I would say because it was always the ball was in our court. So, from the very beginning she [the manager] …always opened up saying if you do ever have any concerns, I am always welcome to hear them… we could feel that we were part of a team, it wasn’t just you by yourself struggling and swimming alone [Susie, B‐RN5].

Other participants recognised the challenging circumstances faced by managers, particularly higher level management, and acknowledged the constraints they encountered in addressing the complexities of a public health pandemic.

I don’t think they were given the tools themselves to help us manage, they were overloaded with extra things from the infection prevention point of view and putting a lot of different things in place… It doesn’t mean they didn’t care; I don’t think they had the capacity because they were given so many extra jobs themselves [Yvonne, B‐NM6].

### 5.2. Connection to Others

#### 5.2.1. Support From Peers

During the crisis, participants created informal support networks, complementing the structured workplace strategies at both sites. Nurses described ‘leaning on each other’ as a vital coping mechanism in their dynamic work environments, with some seeing it as their only option. “W*e were leaning on each other because we felt like there was no other option*.” [A‐RN6]. A number of nurses explained, “I*t was really amongst yourselves that you would have that support, it was more a peer support than anything else”* [Yvonne, B‐NM6].

#### 5.2.2. Teamwork

Collaboration within small teams was seen as a vital and effective approach to managing the uncertainty of the situation. Nurses from both sites highlighted the significance of teamwork and its positive impact on both their well‐being and patient care.

There was a feel of being a team as well, about working with other people… The support that–I guess, even just by being in it together, that you got from your colleagues [Jane, B‐NM3].

### 5.3. Self‐Care Practices

Participants in this study adopted various self‐care strategies in their daily lives to help navigate an uncertain and unfamiliar situation. Many of these strategies centred on self‐enriching activities, such as engaging in creative pursuits and hobbies, while others involved establishing clear personal boundaries. Nurses were able to identify the need to prioritise their own mental and physical health and well‐being in order to care for their patients, colleagues and loved ones.

#### 5.3.1. Self‐Enriching Activities

Many participants emphasised physical exercise and dedicated time to their hobbies. One participant explained:

Work is important, but at the end of the day I think that your self‐worth shouldn’t come from work and so you should invest your time in other things to make you happy. [Susie, B‐RN5].

I tried to exercise as much as possible because I find that a really good release. It’s probably the only time I could go outside [Ivy, B‐NM2].

Many participants emphasised the importance of self‐care strategies, such as reflection, relaxation and prioritising sleep, and made a conscious effort to integrate these practices into their daily routines.

I think I was really quite reflective of my moods, and I started intermittently journaling, which I′d never done before as a way to reflect on work, but also just life in the pandemic [Claire, A‐RN1].

#### 5.3.2. Boundary Setting

Several participants established clear boundaries to safeguard their psychological well‐being, as well as that of their colleagues and team members. These boundaries were self‐imposed and extended beyond their clinical roles to their personal lives. The following quotes illustrate this point well:

Being really clear with my days off. I′ve got like [an] on‐call phone and my personal phone, when I’m off my on‐call phone just left at the hospital or it’s fully off at home [Claire, A‐RN1].

## 6. Discussion

Leadership faces significant challenges in managing unprecedented health crises, such as the COVID‐19 pandemic. Experiences from such events highlight the necessity of strategies that support frontline staff, which must be integrated into preparedness policies [45]. Health leadership literature highlights effective crisis strategies: conveying optimism and vision, deferring to team expertise amid change and leading visibly to foster a flexible, supportive environment [46].

Visible leadership reassures teams through transparency and empathy, building trust during uncertain periods [46]. However, RNs and NMs in this study were critical of the leadership support they received. Contrary to expectations, leadership visibility was lacking, and the support offered felt distant and tokenistic, creating a sense of division between management and frontline staff. Many nurses described feeling abandoned, as leadership seemingly prioritised operational needs and their own well‐being over the growing emotional, physical and mental struggles of the workforce. The long‐term implications of this may be severe, as unresolved burnout can lead to chronic and debilitating effects [47], which is an important consideration for management when adopting interventions to improve retention of workforce.

Proximity of support was highlighted as a key factor in nurses feeling valued and supported. RNs and NMs reported feeling more supported by line managers who were flexible, inclusive and collaborative. Most significantly, they found resilience and coping mechanisms through peer support and a shared sense of *‘being in it together’.* At the onset of the pandemic, there was a strong sense of unity, with everyone ‘*pulling together to get through’*. This collective mentality helped sustain nurses for a time. However, as the demands and pressures of maintaining a workforce under unprecedented and uncertain workloads persisted, cracks began to emerge. Staff expressed concerns that management prioritised service delivery over their mental health, even as they acknowledged the necessity of maintaining healthcare operations. Peer support emerged as a preferred strategy for enhancing team well‐being, offering emotional connection, shared experiences and practical advice. Participants often relied on informal peer networks, which were perceived as more authentic and less stigmatising than formal employee assistance programs [48]. Staff support strategies may need to consider how peer support can be augmented within the organisation. While staff appreciate their peers, NMs may need to consider overt approaches to augmenting this support and connecting management strategies to staff support approaches. While formal employee assistance programs will have a place in supporting the workforce, potential exists to create formal peer support systems that may not exist outside of orientation and transition to practice programs. In a Canadian study, shared leadership approaches were positively associated with creating a healthy work environment and potentially may provide a positive experience of leadership for staff during crisis along with supporting leaders in carrying the burden of supporting staff and each other [49].

While gestures like meal vouchers were appreciated, they lacked depth and longevity. Remarks such as ‘*You know what you’re doing*’ may convey autonomy under normal conditions, but during prolonged stress, they reinforced feelings of isolation and uncertainty. Temporary solutions that fail to consider nurses’ well‐being may be perceived as dismissive, isolating and detrimental to psychological safety, potentially leading to medical errors or unsafe patient conditions [50]. As one nurse explained, *‘We focused a lot more on service delivery than on the well-being of our teams’.* Sustainable, evidence‐informed interventions, such as structured peer programs and brain‐friendly strategies fostering trust and cohesion, are vital to maintain psychological safety and team resilience [51–53], while gratitude practices and interventions are often connected to individual resilience [54]. We note that positive leadership reported in this study was diverse, with some staff feeling like they had received support and others less so. This is compared to a North American study which found transformational leadership was most appreciated during the COVID‐19 crisis [55], but that NMs reported more positive characteristics than their staff did. This is slightly different to our findings, where diverse views were held at all staff levels. Transformational leadership and shared support were associated with better staff well‐being outcomes during the crisis in Belgium as well [56].

Flexibility is a fundamental aspect of nursing, as nurses navigate unpredictable situations daily. They continuously adapt and modify their practice to ensure patients receive high‐quality care in dynamic environments [57, 58]. Throughout their careers, nurses develop essential skills, including critical thinking, communication, time management and emotional intelligence, which enhance their ability to adjust care plans and meet individual patient needs [59, 60]. A key component of this flexibility is teamwork, which enables nurses to pool their resources and strengths to optimise patient outcomes while supporting one another through demanding situations. Shared support and resource sharing, also referred to as social cooperation, was found to significantly strengthen the protective elements of characteristics like grit and a positive attitude on burnout of emergency nurses [61].

In facing unfamiliar challenges, nurses took proactive steps to protect their mental health and well‐being beyond organisational support, using strategies like exercise, journaling, hobbies and disengaging from work phones to maintain balance and normalcy [62–64]. Prioritising sleep and setting boundaries around extra shifts were also key to sustaining their resilience. Despite these personal efforts, few reports indicated that management offered flexible work options, such as shorter shifts, job sharing or self‐rostering, that are known to enhance job satisfaction and retention [65]. While difficult to implement during crises, such adjustments could be supported through educational initiatives and technology to improve uptake and ease managerial concerns [66], particularly in regional settings where fostering leisure and recovery practices is essential.

This study highlights three key insights and implications for health services (Table 3).

**TABLE 3:** Key insights and implications.

Theme	Key insight	Implication for health service
Flexibility and leadership	Frontline innovation thrived under collaborative, adaptive leadership. Authoritarian styles hindered progress.	Develop flexible rostering systems. Develop leadership capacity that is collaborative and consultative to support effective crisis response. Provide managers with training to learn and refine transformational leadership approaches. Enhance visible and collaborative leadership practices.

Peer support	Peer relationships were the most valued form of support, crucial for resilience and well‐being.	Implement structured peer‐support systems. Invest in strengthening team cohesion before crises to ensure a solid support foundation during challenging times.

Proactive self‐care	Staff need self‐care options that are flexible and accessible. Reactive strategies alone are insufficient in crisis contexts.	Embed proactive, well‐resourced self‐care initiatives into organisational culture to promote sustained staff well‐being.

### 6.1. Strengths and Limitations

Strengths of this study include its contextual relevance, capturing nurses’ lived experiences related to an active public health crisis. The use of a pragmatic, exploratory design allowed for flexibility in exploring emergent themes. Adherence to COREQ reporting guidelines ensured methodological transparency. Additionally, the independence of interviewers, external to both the research team and health services, likely enhanced participant openness and reduced response bias.

Limitations include the study’s focus on two regional hospitals in Queensland, which may limit transferability to other healthcare settings. The sample size, while appropriate for qualitative inquiry, restricts broader generalisation. Participants were asked to retrospectively reflect on their experiences, which may have introduced recall bias. Additionally, the absence of demographic diversity may have influenced the range of perspectives captured. Contextual limitations also relate to the culture of healthcare management in Australia at the time of this study, along with the limited number of active COVID‐19 cases Australian healthcare providers had to manage. This context may have a significant influence on the issues identified through participant experiences, and therefore the suggested strategies to implement.

## 7. Conclusion

This study underscores the evolving challenges of a redefined healthcare workplace. In an environment marked by constant change and uncertainty, effective workforce management and adaptable healthcare delivery become imperative. Collaborative and consultative leadership approaches were consistently preferred over authoritarian styles, with a visible leadership presence in the clinical setting emerging as essential. In its absence, a perceived ‘us versus them’ divide between clinicians and management was reported.

The crisis also unlocked new opportunities for flexibility, previously unavailable. Peer support surfaced as the most effective coping strategy, and teamwork proved critical to resilience. Self‐care practices focused on restoring personal well‐being, ‘filling one’s own cup’ and establishing clear boundaries between work and home.

## Funding

This study was supported by the Central Queensland University, RSH/6141. Open access publishing was facilitated by Central Queensland University, as part of the Wiley ‐ Central Queensland University agreement via the Council of Australasian University Librarians.

## Conflicts of Interest

The authors declare no conflicts of interest.

## Data Availability

The data that support the findings of this study are available on request from the corresponding author. The data are not publicly available due to privacy or ethical restrictions.

## Appendix A Interview Questions

For these first five questions, we are interested to understand your experiences of the mental health and well‐being strategies and supports available to you at your workplace.

1. This question has two parts. Can you describe the strategies offered to support your well‐being during the COVID‐19 pandemic at work by…
  a. Your employer/health service?
  b. Your nurse unit manager/line manager or similar?
  Prompt—could you tell me a little more about xx strategy please?
2. How did you feel your employer/NUM/or similar responded to support nurses’ wellbeing and mental health during the pandemic?
  Prompt—too much, too little, overbearing, not relevant?
3. What, if any strategies at work did you use to support your wellbeing throughout the pandemic provided by…
  a. Your employer/health service?
  b. Your ward, NUM, line manager or similar?
  Prompt—could you tell me a little more about xx strategy please?
4. What barriers or challenges, if any, did you experience engaging with any of the support strategies offered to you?
  Prompt—could include ease of access, costly, culturally appropriate etc…Could you tell me a little more about x please?
5. If you did engage with employer provided strategies what were the aspects/factors that helped you engage with and use these services?
  Prompt—could you tell me a little more about xx please?

For these last four questions, we are interested to understand your experiences of any mental health and well‐being strategies and supports you implemented or accessed in your own time—not related to your workplace/employer.

6. How did you manage your mental health and wellbeing providing patient care during the pandemic?
  Prompt—could you tell me a little more about xx please?
7. What other strategies did you use to support your wellbeing during the pandemic?
  Prompt—where these strategies available to you in the workplace?
8. What lessons would you like to share about anything to do with supporting nurses’ wellbeing in a pandemic?
9. Is there anything else you would like to add before we end our interview?
